# Point of Care Testing for the Diagnosis of Fungal Infections: Are We There Yet?

**DOI:** 10.1007/s12281-016-0254-5

**Published:** 2016-04-07

**Authors:** Juergen Prattes, Sven Heldt, Susanne Eigl, Martin Hoenigl

**Affiliations:** Section of Infectious Diseases and Tropical Medicine, Medical University of Graz, Auenbruggerplatz 15, 8036 Graz, Austria; CBmed - Center for Biomarker Research in Medicine, Graz, Austria; Division of Pulmonology, Medical University of Graz, Graz, Austria; Division of Infectious Diseases, Department of Medicine, University of California–San Diego, San Diego, CA USA

**Keywords:** Point-of-care, Diagnosis, Aspergillus, Histoplasma, Cryptococcus, Lateral-flow device, Invasive fungal infections, Diagnostic tools, Fungal infections, Review

## Abstract

Diagnostic tools for invasive fungal infections have continuously improved within the last decades. Nowadays, cultural methods, antigen testing, and molecular tests, such as polymerase chain reaction, are widely used. These methods, however, are accompanied with different limitations as various availability, various turnaround time or high costs. A new generation of point-of-care test has shown promising results in various studies and may overcome some of these limitations. We therefore reviewed the literature for the most promising new point-of-care tests for invasive aspergillosis (*Aspergillus*-specific lateral-flow device test, *Aspergillus* proximity ligation antigen assay), cryptococcosis (cryptococcal lateral-flow assay), and for histoplasmosis (loop-mediated isothermal amplification assay).

## Introduction

Invasive fungal infections (IFIs) are a major cause of morbidity and mortality in immunocompromised patients [1–3]. Host factors such as severe and prolonged neutropenia, allogeneic stem cell transplantation, prolonged use of corticosteroids, prolonged hospitalization at an intensive care unit (ICU), and receipt of recognized T cell immunosuppressants may predispose patients for developing IFI [1, 4].

IFIs have a wide spectrum of clinical presentations, and diagnoses mostly rely on laboratory-based results. Culture-based methods are valuable but limited by time to results, and due to the relatively insensitive for the detection of fungal pathogens [5, 6]. Matrix-assisted laser desorption/ionization time-of-flight mass spectrometry (MALDI-TOF MS) is a very promising development for identification of culture isolates. MALDI-TOF testing is simple to perform, accurate, can technically identify nearly every organism, and may even detect resistance [5]. Nevertheless, impact of MALDI-TOF testing on early diagnosis and treatment of IFIs is limited, as a positive culture result is a prerequisite for that method. Currently, amplification-based assays are under development that may ultimately replace culture-based tests in the laboratory [5]. Amplification-based assays may provide results to the laboratory within 20 min to 5 h. New multiplex polymerase chain reactions (PCRs) are often able to detect a selection of multiple pathogens in a single session [5]. However, currently available amplification assays are mostly limited by relatively high costs, inconsistent performance in terms of sensitivities and specificities, and lack of standardization. Loop-mediated isothermal amplification (LAMP) is a cheaper and more simple technique than traditional PCR, and therefore an advantageous alternative, providing results to the laboratory in less than 60 min [5]. Thus, LAMP for diagnosis of histoplasmosis will be discussed in this review.

Besides molecular diagnostic tools, antigen-based tests are widely used in clinical routine for IFI diagnosis. They are much faster compared to culture and able to provide quantitative results to the clinician optimally within a day [5]. Depending on the setting, however, time to results may vary, depending on factors such as transport to the laboratory that performs the test. In contrast, qualitative antigen-based assays may allow point-of-care (POC) diagnosis of IFI within minutes, frankly on the bedside. Antigen-based POC tests are currently available for *Aspergillus* spp. and *Cryptococcus* spp. [5], and both will be discussed in this review.

## POC Diagnosis of Invasive Aspergillosis

*Aspergillus* spp. and other molds are the major cause of IFIs among patients with underlying hematological malignancies [3, 7]. Successful management of invasive aspergillosis (IA) in these critically ill patients requires early and reliable diagnosis and rapid initiation of appropriate antifungal therapy. A study analyzing autopsy data over two decades has shown in 2013 that rates of premortem diagnosis of IA have increased over the last decade. While 84 % of the IFIs were diagnosed postmortem in the first 5 years of the study, the rate decreased to 49 % in the last 4 years of the study [8]. Most likely reasons for an ongoing increase of premortem IA diagnoses are the introduction of antigen testing in serum and bronchoalveolar lavage fluid (BALF) specimens [9, 10]. Galactomannan antigen (GM) testing is currently considered the gold standard when it comes to biomarkers for IA diagnosis. Several studies have evaluated the performance of GM detection in different specimen (BALF, blood, urine, cerebrospinal fluid) and proposed its utility in daily clinical routine. GM determination, which is performed using the Platelia^™^*Aspergillus* enzyme immunoassay (Bio-Rad, France), however, has some limitations. First, the variable turnaround time within different centers depending on the amount of specimen sent in for GM determination. The Platelia^™^*Aspergillus* enzyme immunoassay (Bio-Rad, France), approved from the FDA as an adjunctive test for IA diagnosis, is performed on a 96-well plate. Second, GM determination requires specially equipped laboratories and trained staff that is not available in all centers. Third, the GM ELISA also detects antigens produced by *Geotrichum capitatum* and cross-reacts with other opportunistic fungal pathogens like *Histoplasma* spp. and may with *Cryptococcus neoformans* [11–13]. Qualitative POC assays for GM detection are currently in development and may be promising for the future, once developed, evaluated, and approved.

### *Aspergillus*-Specific Lateral-Flow Device Test

The POC *Aspergillus*-specific lateral-flow device (LFD) test has been developed more than 5 years ago and evaluated in a number of studies. The LFD is an immunochromatographic assay using a mouse monoclonal antibody, JF5, which binds to an extracellular glycoprotein antigen from *Aspergillus* spp., only secreted during active growth. Minimal required training, simple handling by using BALF samples without any pretreatment, no need for specially equipped laboratories, rapid availability of test results within 15 min, and low costs are the major advantages of the LFD. In case of serum testing, samples need to be pretreated by heating, centrifugation, and adding a buffer solution according to the manufacturer’s recommendations [14, 15]. Results are read by eye after 15-min incubation time and are interpreted depending on the intensity of the test line as negative (−) or weak (+) to strong (+++) positive. Even though the results are read by the naked eye and one may argue that this may lead to poor reproducibility, Wiederhold and colleagues performed a study using a guinea pig model of IA and tested serum and BALF samples in two independent laboratories. Thirty-two of 33 (97 %) serum samples and 26/33 (79 %) BALF samples were in agreement within these two laboratories, indicating a good reproducibility [16]. Cross-reactivities are also rare with the LFD. Only cross-reactions with *Penicillium* spp. are described in the original publication [15]. Since these data were published, interest in this new POC test has increased and several clinical studies evaluated the clinical performance of the LFD test in various patient cohorts and different specimen.

In one of the first in vivo studies evaluating BALF samples from 29 patients with hematological malignancies, the LFD yielded sensitivity of 100 % and a specificity of 81.8 % for diagnosis of IA. Four samples yielded false-positive results, but all of them were interpreted as weak positive only [17]. A further study on BALF-LFD in hematological patients also yielded a 100 % sensitivity and a specificity of 83 % [18]. Even though this study was limited by a very small sample size of hematological patients (*n* = 7), results were similar to the prior mentioned study. The largest study evaluating the performance of the BALF-LFD test in hematological malignancy patients was published in 2015, and 95 BALF samples were analyzed from 72 prospectively enrolled patients of whom 27 patients (30 samples) had probable IA [19]. Per patient sensitivity was lower compared to the former studies (71 %), but specificity was similar (76 %). This may partly explained due to the fact that all patients with false-negative results within the last study were on antifungal prophylaxis/treatment at the time of bronchoscopy. Mold active prophylaxis/treatment may cause a significant decrease of BALF-LFD sensitivity as shown in a recently published study. This retrospective analysis yielded BALF-LFD sensitivity of 86 % in patients not receiving mold-active agents versus 52 % in patients receiving mold-active agents (*p* = 0.006) [20]. In addition, in most studies performed on BALF-LFD, no or little information is given on pretreatment of BALF samples prior to testing. This, however, is of particular interest as pretreatment with dithiothreitol (a commonly used mucolytic agent for respiratory tract samples) containing liquefying agents significantly alters LFD test line intensities and reduces GM levels [21]. In another recently published study by Johnson et al., the performance of real-time PCR, LFD, and GM in BALF samples obtained from patients at risk for IA was evaluated and showed that PCR and LFD had perfect sensitivity of 100 % compared to 87.5 % sensitivity for GM detection [22]. Specificity was slightly lower for both, PCR and LFD, when calculated for each test (87 and 80 %, respectively) but superior to specificity of GM detection with a specificity 66.6 %. PCR and LFD results showed a nearly perfect agreement with a kappa coefficient value of 0.93. Thus, combination of PCR and LFD did not result in an increase of specificity (85.7 %), showing the high potential of the LFD test.

Similar to GM, the LFD may also be performed in serum samples. Results from studies investigating the performance of the LFD when using serum samples, however, showed less promising results compared to BALF testing. This may be in part explained due to the fact that systemic antifungal prophylaxes/therapy may have a stronger influence on serum samples compared to BALF samples [23] which was also shown for GM detection [24]. Held and colleagues reported a sensitivity and specificity of about 40 and 86.8 % for serum-LFD testing in hematological stem cell transplant (HSCT) recipients when only one positive LFD was required for IA diagnoses and a sensitivity of only 20 % and specificity of 97.8 % when two consecutive positive LFD samples were required for diagnosis [25]. Positive samples, however, need to be interpreted in context with clinical signs and symptoms and should trigger further investigations as demonstrated by White and colleagues, who reported a poor positive predictive value (PPV) of 67 % when using serum-LFD results for IA diagnosis but a remarkable increase of the PPV when using LFD in combination with either PCR (100 %) or GM (80 %) for diagnosis [26].

As seen with GM, BALF samples seem to be the most promising when using the LFD test for IA diagnosis in nonneutropenic patients, in particular patient collectives outside of the hematological malignancy setting. A number of studies on BALF-LFD performance in patient cohorts besides hemato-oncological patients have been published, revealing consistent and promising results of the test. In solid organ transplant (SOT) recipients, for example, sensitivities between 91 and 100 % as well as specificities of 80–83 % were reported [17, 27]. Similar performance could be observed for ICU patients with sensitivity of 80 % and specificity of 81 % [28]. The high negative predictive value of 96 % in this study may be used in clinical routine to rapidly rule out suspected IA in ICU patients and may withhold antifungal therapy. This is of particular interest in ICU patients as up to two thirds of ICU patients receiving antifungal agents without evidence for IFIs [29]. Performance of BALF-LFD was also evaluated in patients with underlying pulmonary disease [6]. Sensitivity for IA diagnosis reached 77 % and specificity 92 %. Thus, the LFD showed a significant higher sensitivity compared to mycological culture (77 vs 29 %) and even a higher specificity compared to BALF-GM determination (92 vs 81 %). In contrast, data on the performance of serum LFD testing in nonneutropenic patients, like ICU patients or SOT recipients, are lacking to date.

In conclusion, the LFD test seems to be a promising POC test for IA diagnosis as it showed good performance in clinical studies and overcomes some of the limitations given with GM determination as variable turnaround time, availability, or cross-reactions. Performances of the LFD test in the different patient cohorts as well as in different specimen (using published studies) are displayed in Tables 1 and 2.

**Table 1:** Aspergillus LFD performance in BALF and serum in various patient cohorts

Study	Risk group	Sample size (*n* of patients)	Specimen	Sensitivity	Specificity	Reference
Hoenigl 2012	HM	29	BALF	100	81.8	[17]
Miceli 2015	HM	7	BALF	100	83	[18]
Prattes 2015	HM	72	BALF	71	76	[19]
Johnson 2015	HM and non-HM	32	BALF	100	80	[22]
Hoenigl 2012	SOT	10	BALF	100	80	[17]
Willinger 2014	SOT	47	BALF	91	83	[27]
Eigl 2015	ICU	133	BALF	80	81	[28]
Prattes 2014	Respiratory Disease	221	BALF	77	92	[6]
Held 2013	HSCT	101	Serum	40^a^	86.8^a^	[25]
				20^b^	97.8^b^	
White 2013	HM	103	Serum	81.8^a^	84.8^a^	[26]
				59.1^b^	98^b^	

*HM* hematological patients, *SOT* solid organ transplant recipients, *ICU* intensive care unit, *HSCT* hematological stem cell transplantation recipients, *BALF* bronchoalveolar lavage fluid

^a^Single testing = a minimum of one positive LFD results is required for diagnosis

^b^ Multiple testing = a minimum of two or more positive LFD results are required for diagnosis

**Table 2:** Performance of the BALF *Aspergillus* LFD for probable/proven invasive pulmonary aspergillosis versus no evidence for invasive pulmonary aspergillosis (per BALF sample) in different patient cohorts

Patient group	Sensitivity	Specificity	PPV	NPV
Solid organ transplantation	94 % (15/16)	92 % (89/97)	65 % (15/23)	99 % (89/90)
Intensive care unit	79 % (26/33)	85 % (176/206)	57 % (26/46)	96 % (176/183)
Respiratory diseases	77 % (24/31)	92 % (195/211)	60 % (24/40)	97 % (195/202)
Hematological malignancies	65 % (30/47)	89 % (88/99)	73 % (30/41)	84 % (88/105)

Data derived from published studies: [6, 14, 17–20, 27, 28, 66]

*PPV* positive predictive value, *NPV* negative predictive value

### Proximity Ligation Assay as a Diagnostic Technique for Invasive Aspergillosis

Proximity ligation assays (PLA) are very specific antibody-based tests with high sensitivity and therefore a valuable diagnostic option. Johnson et al. [30] successfully designed a PLA for detecting a *Aspergillus* mannoprotein. They used the monoclonal antibody JF5, which is the same antibody used with the *Aspergillus* LFD test, for targeting a *Aspergillus*-specific extracellular mannoprotein. In their study, *Aspergillus* culture filtrate was gained and spiked to saline and serum [30]. PLA, GM detection by using the Platelia^™^*Aspergillus* enzyme immunoassay (Bio-Rad, France), and the LFD were performed with these samples. The results of the PLA showed a 10- to 100-fold higher sensitivity compared to the GM assay and a 1000-fold higher sensitivity compared to the LFD assay. Furthermore, three BALF samples were tested with the PLA in cases with probable IA and positive GM assay, LFD, and PCR. The PLA results were coherently positive in all three cases [30]. In addition, the PLA assay also had a high specificity and showed no cross-reactivity when tested with culture filtrates of other fungal species (i.e., *Candida*, *Mucor*, *Fusarium*). In conclusion, the *Aspergillus* PLA developed by Johnson et al. seems to be highly sensitive and specific and warrants future investigation in a higher number of clinical samples.

## POC Diagnosis of Cryptococcosis

Cryptococcal meningitis is a life-threatening opportunistic fungal infection caused by *Cryptococcus* spp., primarily by the pathogenic species *Cryptococcus neoformans* var. *grubii* and var. *neoformans* and *Cryptococcus gattii*. Cryptococcosis occurs mostly HIV related [31], but increasing incidence has also been reported in SOT recipients. Overall, cryptococcal meningitis is estimated to affect nearly a million patients per year, with more than 600,000 deaths predominantly in developing resource-limited countries [32]. To reduce the high mortality rate, which reaches 12 % in industrialized countries like the USA [33] and almost 90 % in sub-Saharan and South Africa [34, 35], rapid and reliable diagnostic tools for detection of the basidiomycetous fungi are of utmost importance. Mycological culture is still considered the gold standard but is limited long turnaround time. In addition, appropriately equipped laboratories as well as technical expertise are required for culturing cerebrospinal fluid (CSF), which both are often not available in resource-limited countries. Hence, diagnosis of cryptococcosis mostly relied on direct microscopy of CSF, though this is characterized by poor sensitivity. Therefore, detection of cryptococcal antigen (CrAg), a component of the cryptococcal polysaccharide capsule glucoronoxymannan (GXM), has become increasingly important. To date, two main methods for detection of the CrAg exist. On the one hand, latex agglutination (LA) and on the other hand the enzyme immunoassay (EIA). Both methods are highly sensitive and specific, and less time-intensive compared to culture or microscopy [36]. However, LA as well as EIA have limitations. First, they require laboratory infrastructure and skilled technicians and second, false-negative results and prozone effects (= false-negative results due to very high concentrations of particular analyte in immunoassays) were well known [37].

### Cryptococcal Antigen Lateral-Flow Assay

The CrAg lateral-flow assay (LFA) is a POC test for the detection of CrAg, which was developed by Immy Inc. (Norman, OK) in 2009. The CrAg LFA satisfies all requirements for a POC test: low costs (approximately 2$ per strip in resource limited settings), excellent test performance, easy to use and rapid test results (available in 10 min) [38]. Moreover, the CrAg LFA is temperature stable and cross-reactions with other fungi are rare. For testing, 40 μl of body fluid (serum, plasma, urine, and CSF) without pretreatment are applied to a reservoir of the LFA test strip. If GXM is present, the anti-GXM monoclonal antibodies—the test uses two monoclonal antibodies and can recognize all four GXM serotypes (A–D)—forms a visible line. The handling of the test and the interpretation of the test line result have been well demonstrated in publications [39, 40]. In one study, sensitivity of the serum CrAg LFA of 100 % and a specificity of 99.8 % was reported, when using serum LA test as gold standard [41]. In the same year, another study also reported a 100 % sensitivity of the serum CrAg LFA which was higher than the 91 % found for serum LA. Importantly, this study included only very few patients with HIV infection, indicating the potential diagnostic value of the CrAg LFA also for other populations with cryptococcal disease [42]. Other studies comparing the CrAg LFA to other CrAg based tests in low-income countries show similar results: sensitivities of 95.6–100 % and specificities of 96.9–99.5 % when using serum specimens [43, 44], and sensitivities of 80–100 % and specificities of 73.8–91.5 % when using urine samples (also depending on the diluents) [45]. The reliable performance of the CrAg LFA was confirmed in studies evaluating its performance compared to culture or composite reference standard: sensitivities of 98.3–100 % in serum samples, 92–98 % in urine specimens, and 86.1–100 % in CSF samples [44, 46–48]. Since 2011, the World Health Organization (WHO) has added the CrAg LFA to the LA test as preferred method for diagnosis of cryptococcal disease [49].

To summarize, the CrAg LFA has several advantages: multiple samples can be run simultaneously, cost-effective, and do not need electricity, specially equipped laboratory, or skilled technicians. Importantly, CrAg LFA is not useful to check treatment response, as the clearance of CrAg is a slow and also independent process that devitalize the yeast [50, 51]. During effective antifungal therapy, CrAg LFA titers may therefore remain elevated [52, 53].

CrAg has been confirmed as a reliable predictor of development of cryptococcal disease after initiating antiretroviral therapy (ART), and even mortality [47, 54–56]. Twenty to 30 % of patients in resource-poor countries present cryptococcal meningitis within 3 months after initiating ART [57, 58]. French et al. reported that CrAg may be detectable as early as 100 days before the beginning of symptomatic cryptococcal disease [59]. Therefore, the WHO recommends routine serum or plasma CrAg screening in HIV-positive patients without ART and a CD4 cell count <100 cells/mm^3^ [49]. With regard to the role of the test in preemptive screening, one important questions remains to be answered: how to proceed with patients that do not have symptoms of cryptococcal meningitis but a positive CrAg result? While the WHO recommends a “screen and treat asymptomatic positives with fluconazole” strategy, future studies are needed to answer this question.

In conclusion, the diagnostic accuracy of CrAg has been evaluated in multiple studies in resource-rich and resource-limited countries and showed excellent sensitivity and specificity on serum, plasma, urine [44, 48], and CSF samples [47]. A positive CrAg allows the treating physician to initiate appropriate antifungal therapy without delay and may additionally predict mortality risk. CrAg LFA may also play a future role in preemptive screening, consequently in cost reduction and outcome improvement [45, 60–62]. This POC test, therefore, clearly has the potential to markedly improve the early diagnosis of cryptococcosis.

### Advancement in the Diagnosis of Histoplasmosis

Histoplasmosis and especially progressive disseminated histoplasmosis (PDH) in patients with immunodeficiency is caused by *Histoplasma capsulatum*, a fungus endemic in North and South America, Africa, Asia, and Australia [63]. Diagnosis is challenging; in particular, the current alternatives to culture diagnostics are too expensive for resource-challenged countries [64]. Rapid and reliable assays are important for the treatment of PDH, as mortality rate increases up to 42 % when therapy is delayed and up to 95 % when PDH is misdiagnosed [64]. The diagnostic process is also complicated as symptoms of PDH are unspecific and similar to infections with *mycobacteria* or *leishmania* [65]. The currently available diagnostics for *H. capsulatum* comprise detection by culture, which can be enhanced by culture identification with the AccuProbe® test. The main problem with this method is that culturing *H. capsulatum* can take a few weeks, and the AccuProbe® test is expensive [64]. As an alternative, there are different PCR-based assays available. However, these are also expensive and not well evaluated yet [65].

Scheel et al. [64] conducted a pilot study in which they developed a LAMP assay and evaluated its sensitivity and specificity in comparison to the PCR-based assays. LAMP is an alternative method for DNA amplification using a different polymerase (*Bst*) as used for traditional PCR (*Taq*). *Bst*-polymerase is cheaper and more robust than *Taq*. LAMP also requires less expensive equipment compared to traditional PCR. In their study, Scheel et al. [64] chose the *Hcp100*-locus of *H. capsulatum* for amplification and designed a LAMP primer, as that locus has only few similarities to related microorganisms, and there are no any known mutations that may lead to false negativity. Furthermore, they collected different geographic subspecies of *H. capsulatum* isolates (*n* = 91), extracted the DNA, and proceeded with PCR and LAMP to compare the sensitivities of these two assays, and to evaluate the limit of necessary genomes for the LAMP assay to become positive (limit of detection (LOD)). In addition, Scheel et al. [64] collected urine samples from healthy persons (*n* = 10) as well as from HIV patients with PDH (*n* = 6; proven by clinical symptoms, positive antigen detection and positive culture with urine samples), where they compared sensitivity and specificity of PCR and LAMP. The LOD was noted to be a median of 6 genomes (from 1 to 30 genomes, strain-dependent), and 10-fold lower than the LOD of PCR. Within an incubation time of 1.5 h, no cross-reactivity could be recognized when the designed LAMP primer had been used for assays with other fungi, *M. tuberculosis* or human DNA, which means a specificity of 100 % in this pilot study. Coherent to these findings, there was no false-positive LAMP assay when testing the urine samples of the healthy persons. LAMP was only able to detect *H. capsulatum* DNA in four of the six urine samples (67 %), which was still superior to the simultaneously conducted PCR assays, and was not able to show any positive test result when testing the urine samples. In conclusion, Scheel et al. [64] showed that *H. capsulatum* DNA can be detected from cultured isolates as well as from urine samples of patients with PDH when using their LAMP assay. As the search for a POC test for *H. capsulatum* continues, the study presents interesting findings that may advance diagnosis in the meantime.

## Conclusion

Important advances have been made during the last years in particular with regard to POC diagnosis of cryptococcosis but also for IA. The CrAg LFD not only has excellent sensitivity over all four serotypes of *Cryptococcus* but is also FDA approved and commercially available; commercial availability of the *Aspergillus* LFD is still pending. While LAMP may offer significant advancement in the diagnosis of histoplasmosis, the search for reliable POC tests for other fungi continues.
